# Creatine supplementation enhances muscle force recovery after eccentrically-induced muscle damage in healthy individuals

**DOI:** 10.1186/1550-2783-6-13

**Published:** 2009-06-02

**Authors:** Matthew B Cooke, Emma Rybalka, Andrew D Williams, Paul J Cribb, Alan Hayes

**Affiliations:** 1 Exercise Metabolism Unit, Centre for Ageing, Rehabilitation, Exercise and Sport, School of Biomedical and Health Sciences, Victoria University, Melbourne, Australia; 2 Exercise & Sport Nutrition Lab, Center for Exercise, Nutrition and Preventive Health, Department of Health, Human Performance & Recreation, Baylor University, Waco, Texas, USA; 3 School of Human Life Sciences, University of Tasmania, Launceston, Tasmania

## Abstract

**Background:**

Eccentric exercise-induced damage leads to reductions in muscle force, increased soreness, and impaired muscle function. Creatine monohydrate's (Cr) ergogenic potential is well established; however few studies have directly examined the effects of Cr supplementation on recovery after damage. We examined the effects of Cr supplementation on muscle proteins and force recovery after eccentrically-induced muscle damage in healthy individuals.

**Methods:**

Fourteen untrained male participants (22.1 ± 2.3 yrs, 173 ± 7.7 cm, 76.2 ± 9.3 kg) were randomly separated into 2 supplement groups: i) Cr and carbohydrate (Cr-CHO; n = 7); or ii) carbohydrate (CHO; n = 7). Participants consumed their supplement for a period of 5 days prior to, and 14 days following a resistance exercise session. Participants performed 4 sets of 10 eccentric-only repetitions at 120% of their maximum concentric 1-RM on the leg press, leg extension and leg flexion exercise machine. Plasma creatine kinase (CK) and lactate dehydrogenase (LDH) activity were assessed as relevant blood markers of muscle damage. Muscle strength was examined by voluntary isokinetic knee extension using a Cybex dynamometer. Data were analyzed using repeated measures ANOVA with an alpha of 0.05.

**Results:**

The Cr-supplemented group had significantly greater isokinetic (10% higher) and isometric (21% higher) knee extension strength during recovery from exercise-induced muscle damage. Furthermore, plasma CK activity was significantly lower (by an average of 84%) after 48 hrs (P < 0.01), 72 hrs (P < 0.001), 96 hrs (P < 0.0001), and 7 days (P < 0.001) recovery in the Cr-supplemented group.

**Conclusion:**

The major finding of this investigation was a significant improvement in the rate of recovery of knee extensor muscle function after Cr supplementation following injury.

## Background

Exercise-induced skeletal muscle injury is well understood as the product of unfamiliar or strenuous physical activity, and eccentric (lengthening) contractions under high loads are primarily responsible [1,2]. Eccentric exercise leads to the disruption of the normal muscle ultrastructure and alters sarcolemmal and sarcoplasmic reticulum (SR) function which results in an increase in intracellular calcium and subsequent activation of degradative pathways [3]. The trauma created by this type of exercise initiates a myriad of events that lead to reductions in muscle force, increased soreness, and impaired muscle function [1,2]. Therefore, strategies that may reduce the negative effects of eccentric exercise and/or promote the regenerative processes would benefit athletes and others that perform strenuous/unaccustomed physical activity.

One dietary supplement that may reduce the severity of exercise-induced muscle damage and/or promote recovery is creatine monohydrate (Cr) (n [aminoiminomethyl]-N-methylglycine). Although well-over 200 studies have examined Cr's ergogenic potential in the last 20 years [4], few studies [5-8] have directly examined the effects of Cr supplementation on indicators of muscle damage and recovery after damage. Rawson and colleagues [7] supplemented male subjects with Cr for 5 days prior to 50 maximal eccentric contractions. The study showed that maximal isometric force of the elbow flexors, and serum creatine kinase (CK) and lactate dehydrogenase (LDH) activity, in response to eccentric exercise were not significantly different between the Cr-supplemented and control groups during the 5 days following exercise. Therefore, it was suggested that Cr supplementation does not reduce indirect markers of muscle damage or enhance recovery from high-force eccentric exercise. Similarly, Warren *et al*. [8] demonstrated that recovery of mouse anterior crural muscle strength after damage (induced by 150 eccentric contractions) was unaffected following 2-weeks of Cr supplementation. Following 3 minutes recovery, there was no effect on isometric strength or on torque loss at any eccentric or concentric angular velocity. However, a number of limitations exist with this study. Firstly, researchers were only interested in how increased muscle Cr influenced peak strength loss and not the recovery of strength *per se *after injury. Therefore, the 3 min recovery period may not be long enough to see any beneficial effect of Cr supplementation on muscle strength loss. Secondly, Cr supplementation may have attenuated other markers of muscle damage such as blood concentrations of myocellular proteins. However, since injury assessment was only muscle function based, these were not measured.

The effect of Cr supplementation upon inflammatory and muscle soreness markers has also been examined following prolonged running [5]. Experienced marathon runners were supplemented (4 doses of 5 g of Cr) for 5 days prior to a 30 km race. Blood samples were collected pre-race, and 24 hours following the end of the test, to measure for CK, LDH, prostaglandin E2 (PGE2) and TNFalpha (TNF-α). Athletes from the control group presented an increase in all muscle soreness markers, indicating a high level of cell injury and inflammation, while Cr supplementation significantly attenuated these increases, with the exception of CK. However, while this Cr supplementation protocol may be an effective strategy in maintaining muscle integrity during and after intense prolonged aerobic exercise, it may not be sufficient to protect muscle fibres from more damaging exercises, such as those shown by Rawson *et al*. [7].

Therefore, the purpose of this investigation was to supplement a group of healthy participants with either Cr or a placebo prior to, and in the days after a single bout of eccentric exercise. The extent of, and recovery from, damage was evaluated by the following established, indirect markers of exercise-induced muscle damage; knee extension/flexion force development (MVC), and plasma CK and LDH activity [9,10]. We hypothesized that supplementation with Cr would reduce muscle damage and restore muscle strength earlier after a single bout of eccentric exercise.

## Methods

### Participants

Fourteen healthy untrained males (22.1 ± 2.3 yrs, 173 ± 7.7 cm, 76.2 ± 9.3 kg) volunteered for this study. Descriptive characteristics of the participants are presented in table 1. To meet the criteria the men (a) were non-smokers; (b) had not participated in resistance-training, or any form of structured exercise, for at least six months; (c) had not ingested any ergogenic supplement for a 24-week period prior to the start of supplementation; and (d) agreed not to ingest any other nutritional supplements, or non-prescription drugs that may affect muscle re-growth during the study. In addition, participants agreed to refrain from using any remedy (i.e. massage, ultrasound etc.) for muscle soreness other than consumption of the supplement given; and agreed not to participate in any form of physical activity 2 weeks prior to supplementation and during the 2 week recovery period. All participants were informed verbally, as well as in writing, as to the objectives of the experiments, together with the potential associated risks. All participants signed an informed consent document approved by the Human Research Ethics Committee of Victoria University of Australia. All procedures conformed to National Health and Medical Research Council guidelines for the ethical conduct of research involving humans.

**Table 1:** Participant baseline characteristics

Characteristics	CHO	Cr-CHO	P-value
Age (yrs)	21.7 ± 3	22.6 ± 2	0.52
Weight (kg)	74.4 ± 7	77.9 ± 12	0.51
Leg Press 1 RM (kg)	85.9 ± 16	83.62 ± 15	0.80
Leg Extension 1 RM (kg)	40 ± 10	36.4 ± 10	0.49
Leg Flexion 1 RM (kg) Extension	26.8 ± 16	34.1 ± 13	0.35

Data are means ± standard deviations of mean. SI unit conversion factor: 1 kg = 2.2 lbs

### Experimental design

All procedures were completed at the Human Performance Laboratory at Victoria University. Two weeks prior to baseline testing, participants underwent 1 repetition maximum (RM) strength assessments on the dominant limb and a familiarisation session of the equipment that would be utilized to assess muscle performance. The dominant limb would undergo the damage protocol, while the contralateral limb served as the control. Participants were randomised in a double-blind placebo-controlled fashion into 2 groups: carbohydrate-only (CHO) (n = 7) or Cr-carbohydrate (Cr-CHO) (n = 7), and issued with their supplement and dosing instructions. On day 1, participants arrived at the laboratory in the morning and underwent baseline performance assessments and blood sampling. Participant's then underwent catherization of the forearm vein and performed an exercise session designed to cause damage to the knee extensor and flexor muscles. Blood samples were taken at 30 minutes, 1, 2, and 4 hours following the bout of exercise. The participants were instructed to return to the laboratory 24 hours post-exercise, and again at the same time on day 2, 3, 4, 7, 10 and 14 for further blood sampling and all muscle performance assessments. All participants arrived at all assessment days in (8 hour) fasted state.

### Dietary Supplementation

The supplements were provided to the participants in identical, unmarked, sealed containers, supplied by AST Sports Science, Golden, Colorado USA. For five days prior to the bout of exercise, the Cr-CHO group consumed a supplement (1.5 g^-1 ^kg of body weight^-1 ^day) that provided a loading dose of Cr (0.3 g^-1 ^kg of body weight^-1 ^day). This provided a 70 kg participant with approximately 21 g of Cr^-1 ^day, with the remainder (84 g in this case) being CHO in the form of glucose. The participants were shown how to consume this dose in several smaller servings each day, i.e., 20–30 g of supplement mixed in water and consumed immediately, once with breakfast, lunch, in the afternoon and after the evening meal. This procedure has been reported to consistently increase muscle Cr concentrations [11]. The CHO group consumed an equivalent per body weight dose of CHO (glucose) only. After the bout of exercise, participants were instructed to take one serving of a supplement (0.5 g^-1 ^kg of body weight^-1 ^day) that provided the Cr-CHO group with a maintenance dose of Cr (0.1 g^-1 ^kg of body weight^-1 ^day) during the 14-day recovery period. Again, the remainder of the supplement was CHO in the form of glucose, and the CHO groups ingested an equivalent per body weight dose of glucose only.

Participants' diets were monitored and assessed as previously described by this laboratory [12]. In brief, participants were shown how to record their dietary habits in diaries provided. During the final recovery week each participant submitted a 7-day written dietary recall (consisting of 5 week days and two weekend days) for the calculation of macronutrient and energy intake. Mean energy intake is expressed in kcal^-1 ^kg of body weight per day; protein, fat and carbohydrate are expressed in g^-1 ^kg of body weight per day. The participants were asked to report any adverse events from the supplements in the nutrition diaries provided. No adverse events were reported by the participants.

### Resistance exercise protocol

Two weeks prior to the session, unilateral (dominant limb) concentric 1 RM assessments were completed for each participant using a procedure prescribed by the National Strength and Conditioning Association (NSCA) [13]. An NSCA certified Strength and Conditioning Specialist supervised all lifts and the damage protocol completed by participants. The resistance exercise session was designed to cause muscle damage. Using a modified version of a procedure previously described [14,15], the workout consisted of three exercises; 1) leg press; 2) leg extension and 3) leg curl (Universal, Cedar Rapids, IA, USA) utilizing 120% of the participants' predetermined concentric 1 RM for each exercise. The participant was required to lower the weight by themselves through the entire range of motion (ROM) at a predetermined cadence (4 seconds) given verbally. This constituted 1 repetition. The participant completed 40 eccentric-only repetitions (4 sets × 10 with 3 minutes rest between sets) of each exercise in this manner. All participants were verbally encouraged during each set to maintain the required lowering speed. However, if the participant was not able to do this in the later stage of the set, (as a result of fatigue), then a brief (5–15 second) pause between the last 2–3 repetitions was permitted. Although the workout was extremely difficult, all participants were able to complete the protocol as outlined.

### Performance assessments

Muscle performance before and after the bout of eccentric exercise was measured by voluntary isokinetic knee flexion and isokinetic/isometric knee extension of each leg using Cybex™ Testing and Rehabilitation System (Cybex International Inc. Ronkonkoma, New York). A protocol similar to that described by [16] was utilized. Measurements of isokinetic knee extension and flexion torque were performed at 60°/s (1.57 rad.s^-1^) velocity torque in one continuous kicking motion. ROM for knee extension and flexion was from 90° to 0° and 0° to 120°, respectively (0° = full knee extension). Maximal isometric strength was determined in three contractions at a knee angle of 60° and of 5-s duration. There was a 20 second rest between each isometric contraction, and a 60 second rest between the isokinetic and isometric force measurements.

Strength values obtained from Cybex tests were expressed as percentage of pre-exercise values and normalized to contralateral controls. Previous research has shown this to be a successful means of reporting muscle strength and performance data, and removes any improvement in muscle performance recovery of the injured limb due to familiarization of the test [16,17]. Test, retest reliability trials were completed on the Cybex dynamometer prior to this study and provided a coefficient of variance (CV) of less than 5% for each parameter measured.

### Blood Sampling

Approximately 10 mls of venous blood was sampled from the antecubital fossa vein via catheterisation before and after the bout of eccentric exercise on day 1. Venipuncture technique was used to draw further blood samples at 2, 3, 4, 7, 10 and 14-days after the resistance exercise session. The blood was immediately placed into an ethylediniaminetetra-acetic acid (EDTA) tube, inverted and rolled, then transferred into eppendorf tubes and centrifuged at 3000 rpm for 15 min at 4°C. Plasma was removed and aliquoted into labelled eppendorf tubes and stored at -80°C for subsequent analysis of CK and LDH activity. For CK, plasma samples were analysed by a 2-step enzymatic colorimetric process using a VITROS 750 Chemistry System according to the method of [18]. For LDH activity, plasma samples were analysed using a single step enzymatic rate process requiring readings on a UV-visible spectrophotometer (SHIMADZU UV-1700, SUZHOU Instrumental manufacturing Co. Ltd, China) according to the method of [19].

### Statistical Analysis

Participant characteristics are reported as means ± SD. All other values are reported as means ± SE. Muscle performance data was expression as a percentage of baseline values. Muscle performance variables were analyzed using 2 × 7 (group × day [Day 1, 2, 3, 4, 7 10 and 14) repeated measures ANOVA to effectively assess the changes in muscle function/strength following supplementation post exercise. Blood variables were analyzed using 2 × 14 (group × day [baseline, 30 min, 60 min 2 hours, 4 hours, day 1, 2, 3, 4, 7 10 and 14) repeated measures ANOVA to effectively assess the changes in markers of muscle damage following supplementation post exercise. LSD pairwise comparisons were used to analyze any significant group × time interaction effects. Baseline variables, total work performed during the resistance exercise session and dietary intake between groups was analyzed using an independent students' t-test. An alpha level of 0.05 was adopted throughout to prevent any Type I statistical errors.

## Results

### Participant Characteristics

At baseline there were no differences in the age, body weight or strength level (1 RM) between the two groups (Table 1).

### Resistance Exercise Session (Total Work)

No differences in total work performed during the resistance exercise session were observed between the two groups (Table 2).

**Table 2:** Resistance Exercise Session (Total Work)

Characteristics	CHO	Cr-CHO	P-value
Leg Press 1 RM (kg)	103 ± 16	100 ± 11	0.81
Leg Extension 1 RM (kg)	48 ± 9	44 ± 5	0.44
Leg Flexion 1 RM (kg) Extension	32 ± 9	41 ± 6	0.36

Data are means ± standard deviations of mean. SI unit conversion factor: 1 kg = 2.2 lbs

### Dietary Analysis

One-week dietary analysis (excluding supplementation) revealed no differences in energy, protein, fat and carbohydrate intake between groups throughout the study (Table 3).

**Table 3:** Dietary Analyses

	CHO	Cr-CHO	P-value
Energy (kcal·kg·d^-1^)	32.7 ± 3.9	33.3 ± 4.6	0.80
Protein (g·kg^-1 ^d^·-1^)	0.92 ± 0.09	0.91 ± 0.13	0.77
Fat (g·kg^-1^·d^-1^)	0.92 ± 0.18	1.08 ± 0.18	0.12
Carbohydrate (g·kg^-1^·d^-1^)	4.33 ± 1.00	4.93 ± 0.81	0.24

Data are means ± standard deviations of mean. SI unit conversion factor: 1 kcal = 4.2 kJ

### Muscle Strength and Performance Assessment

#### Isometric Knee Extension Strength

**Figure 1:**
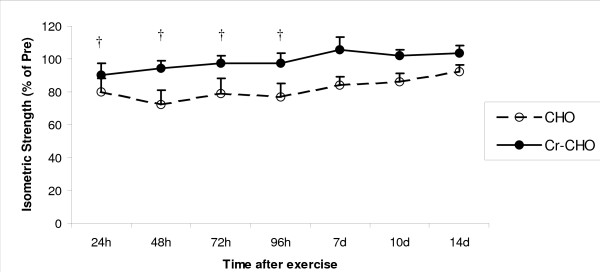
**Effect of CHO and Cr-CHO on isometric knee extension muscle strength after exercise-induced muscle damage**. Data (mean ± SE) represents isometric knee extension muscle strength expressed as a percentage of pre-exercise strength taken during the 14 days recovery. † represents (p < 0.05) difference between groups.

Pre-exercise absolute values for isometric knee extension strength were 234 ± 24 Nm and 210 ± 11 Nm for the CHO and Cr-CHO groups, respectively. No differences were detected. A significant main effect for time was observed in muscle strength following the resistance exercise session indicating reductions in strength (expressed as a percentage of pre-exercise strength) in both groups persisted for 14 days (P < 0.05). A significant main effect for group (P < 0.01) and group × time interaction (P < 0.05) was observed in muscle strength following the resistance exercise session, suggesting that although reductions in strength were also observed in the Cr-CHO supplemented, these reductions were a far smaller % than observed in the CHO group (Figure 1). Indeed subsequent post-hoc analysis revealed significantly higher muscle strength at 24 hours (P < 0.05), 48 hours (P < 0.01), 72 hours (P < 0.05) and 96 hours (P < 0.05) in the Cr-CHO group compared to CHO supplemented group (Figure 1.)

#### Isokinetic Knee Strength

**Figure 2:**
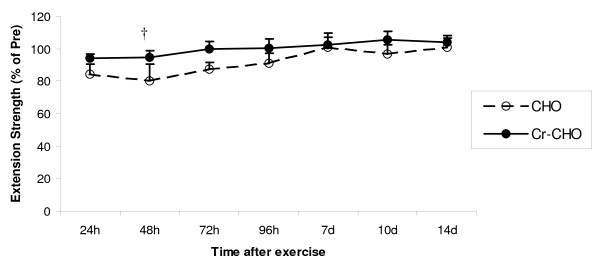
**Effect of CHO and Cr-CHO on isokinetic knee extension muscle strength after exercise-induced muscle damage**. Data (mean ± SE) represents isokinetic knee extension muscle strength expressed as a percentage of pre-exercise strength taken during the 14 days recovery. † represents (p < 0.05) difference between groups.

Pre-exercise absolute values for isokinetic knee extension strength were 206 ± 13 Nm and 197 ± 10 Nm for the CHO and Cr-CHO supplemented groups, respectively. No differences were detected. A significant group × time interaction was observed in isokinetic knee extension strength during recovery (P < 0.05), with subsequent post-hoc analysis revealing that the Cr-CHO supplemented group had higher isokinetic knee extension peak torque compared to the CHO group at 48 hours post resistance exercise (P < 0.05, Figure 2.).

**Figure 3:**
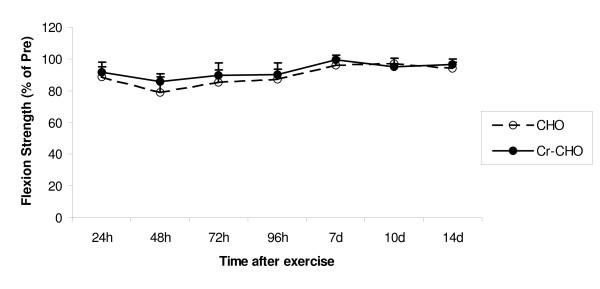
**Effect of CHO and Cr-CHO on isokinetic knee flexion muscle strength after exercise-induced muscle damage**. Data (mean ± SE) represents isokinetic knee flexion muscle strength expressed as a percentage of pre-exercise strength taken during the 14 days recovery.

Pre-exercise absolute values for isokinetic knee flexion strength were 135 ± 9 Nm and 123 ± 9 Nm for the CHO and Cr-CHO groups, respectively. No statistically significant interactions were observed across groups (Figure 3).

#### Plasma Enzyme Activity

**Figure 4:**
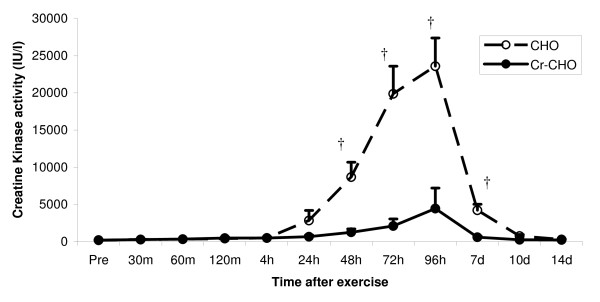
**Effect of CHO and Cr-CHO on plasma CK activity after exercise-induced muscle damage**. Data (mean ± SE) represents plasma CK activity (IU/l) taken during the 14 days recovery. † represents (p < 0.05) difference between groups.

Pre-exercise CK activity was 176.1 ± 59.2 IU·1^-1 ^and 196.4 ± 37.9 IU·1^-1 ^(mean ± SEM) in the CHO and Cr-CHO groups, respectively. No significant differences were detected. Figure 4. illustrates a significant main effect for time (P < 0.0001) for CK activity following the resistance exercise session. Subsequent post-hoc analysis showed CK activity to be significantly elevated above baseline at 48 hours (P < 0.0001), 72 hours (P < 0.0001) and 96 hours (P < 0.0001) post-exercise. A trend towards significance was observed at day 7 (P = 0.074). A significant main effect for group (P < 0.0001) and group × time (P < 0.001) interaction was observed in plasma CK activity, indicating that participant CK response was not similar, in terms of magnitude, at all recovery time points following the resistance exercise session (Figure 4). Indeed, subsequent post-hoc analysis revealed significantly lower plasma CK activity at days 2 (P < 0.01), 3 (P < 0.001), 4 (P < 0.0001), and 7 (P < 0.001) post-exercise in the Cr-CHO compared to CHO group, after which CK activity in the plasma had returned to baseline in both groups (Figure 4).

**Figure 5:**
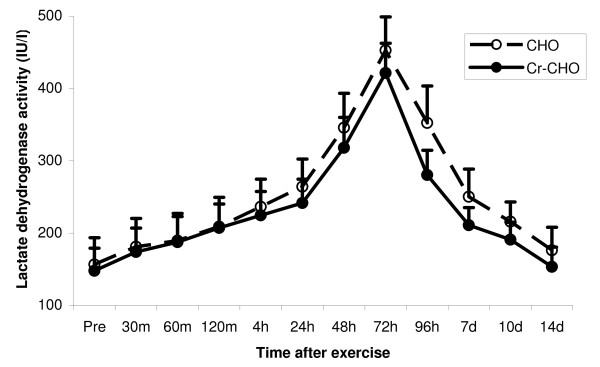
**Effect of CHO and Cr-CHO on plasma LDH activity after exercise-induced muscle damage**. Data (mean ± SE) represents plasma CK activity (IU/l) taken during the 14 days recovery.

Pre-exercise LDH activity was 156.6 ± 37.1 IU·1^-1 ^and 148.0 ± 31.3 IU·1^-1 ^(mean ± SEM) in the CHO and Cr-CHO supplemented group, respectively. No significant differences were detected. Similar to CK, a significant main effect for time (P < 0.0001) was observed for LDH activity following the resistance exercise session, with subsequent post-hoc analysis showing LDH activity to be significantly elevated above baseline at 24 hours (P < 0.01), 48 hours (P < 0.0001), 72 hours (P < 0.0001), 96 hours (P < 0.0001) and at day 7 (P < 0.05) post-exercise. However, the increases in LDH were far lower than for CK, such that only a trend towards a main effect for group was observed (P = 0.093), although this still indicates that plasma LDH activity was generally lower in the Cr-CHO supplemented group compared to the CHO group (Figure 5).

## Discussion

The primary objective of this study was to determine whether consumption of Cr prior to, and following exercise-induced damage, improves force recovery and markers of muscle damage in healthy individuals. Following repeated eccentric exercises, isokinetic knee extension and flexion and isometric knee extension peak torque was significantly reduced, and remained significantly lower than pre-exercise values, for approximately 4 days or longer. Importantly, isometric (21% higher) and isokinetic (10% higher) knee extension strength were both significantly greater during recovery with consumption of a Cr-CHO supplement compared to a supplement with CHO alone.

The observed decrements in muscle strength were in accordance with previous studies, with Brown and colleagues [14] showing similar reductions, although others demonstrated less reductions in strength [7,17]. Such varying responses in the magnitude of strength loss following eccentric exercises are possibly due to the different muscle groups used (i.e. elbow flexors of the forearm vs. knee extensor/flexors muscles groups) and/or the protocol utilized to induce muscle damage [7,17,20]. It should also be noted that muscle strength was expressed as a percentage of pre-exercise strength values and normalised to contralateral (undamaged) controls. This is a common method of analysing loss of muscle strength following exercise-induced damage [7,14,17], which therefore not only normalises data by accounting for any improvements during the recovery period as a result of familiarisation, but more importantly reduces the inter-individual variability in muscle strength between participants.

Extensive literature has examined the effects of Cr supplementation on exercise performance, in particular high intensity exercise [21]. However, only a few studies have investigated the efficacy of Cr supplementation on muscle recovery after injury [5-8]. In 2001 and 2007, Rawson and colleagues examined the effects of Cr supplementation on muscle damage and recovery following 2 different exercise intensities; a high-force, eccentric exercise [7] and a low force, hypoxic resistance exercise challenge [6]. In the first study, male participants were supplemented with Cr for 5 days prior to 50 maximal eccentric contractions. Results showed no significant differences in maximal isometric force of the elbow flexors, or serum CK or LDH activity, between the Cr-supplemented and dextrose control group during the 5 days post-exercise [7]. In the second study, male participants were supplemented with Cr for 5 days prior to, and 5 days following a squat exercise protocol (5 sets of 15–20 repetitions at 50% of 1 repetition maximum [1 RM]). Similar to the first study, oral Cr supplementation had no effect on reducing the extent of muscle damage and/or enhancing the recovery following the resistance exercise challenge [6].

In the current study however, the Cr-supplemented group exhibited an enhanced rate of muscle function recovery compared to the placebo group; as evident by the higher muscle strength values for both the isometric and isokinetic knee extension during the recovery period following exercise-induced muscle damage. Such differing observations could be in part due to the length of supplementation period and/or post-exercise supplementation. In the first study by Rawson and colleagues (2001), participants were only supplemented for 5 days prior to the exercise-induced damage protocol; with no continuation of supplementation following the exercise bout [7]. Willoughby and Rosene [22] have suggested that by continuing Cr supplementation after a resistance exercise bout (initial stimulus), Cr may act as a co-regulator, or direct manipulator of gene transcription of amino acid pools, thus enhancing myofibrillar protein synthesis during the recovery period post-injury. Indeed Olsen et al. (2006) supported such a suggestion by recently demonstrating for the first time in human skeletal muscle fibres that Cr supplementation amplifies the training-induced increase in satellite cell number and myonuclei concentration [23], and thus potentially, muscle regeneration.

Although Cr supplementation was continued following the exercise bout in the second study by Rawson and colleagues [6], it is possible that the resistance exercise session, which was designed to be hypoxic in nature, as opposed to high force, eccentric exercise, may not have elicited enough muscle damage to unmask the anabolic effects of Cr supplementation [24]. Taken together, it is evident from the current study that Cr supplementation prior to, and during recovery from, an eccentric exercise resistance training session, provides faster recovery in strength. It is not uncommon for resistance trained athletes to undertake subsequent training sessions 2 to 3 days following a previous training session. Such an increase in strength output during recovery would presumably allow for a higher training load during subsequent training sessions in the days following the initial exercise bout. Indeed, this may be one of the explanations behind greater mass and strength gains observed in resistance trained participants ingesting Cr-containing supplements [25].

While the majority of studies have examined the role of Cr during the recovery period post exercise [25-27]; a number of studies have suggested a possible beneficial role during exercise [28-30]. The sarcoplasmic reticulum (SR) Ca^2+^pump derives its ATP preferentially from PCr via the CK reaction [28]. Local rephosphorylation of ADP by the CK-PCr system maintains a low ADP/ATP ratio within the vicinity of the SR Ca^2+ ^pump and ensures optimal Ca^2+ ^pump function (i.e. removal of calcium from the cytoplasm) [31]. However, when rates of Ca^2+ ^transport are high (as seen in muscle damage), there is a potential for an increase in [ADP], thus creating a microenvironment (i.e. high [ADP]/[ATP] ratio) that is unfavourable for ATPase function, and as a consequence, SR Ca^2+ ^pump function may be diminished [28,31]. Furthermore, a decrease in [PCr] below 5 mM, which is characteristic of this increased ATPase activity; reduces local ATP regeneration potential of the CK/PCr system [29,30]. Thus, by supplementing with Cr prior to, but also following exercise-induced muscle damage, PCr concentrations within the muscle will be increased, and therefore could theoretically improve the intracellular Ca^2+ ^handling ability of the muscle by enhancing the CK/PCr system and increase local rephosphorylation of ADP to ATP, thus maintaining a high [ATP]/[ADP] within the vicinity of SR Ca^2+^-ATPase pump during intense, eccentric exercise. However, this concept requires further investigation.

Myofibrillar enzymes CK and LDH are widely accepted as markers of muscle damage after prolonged exercise [32-34]. Due to the different clearance rates of these enzymes, plasma CK and LDH were measured at 1, 2, 3, 4 hours following exercise and on days 1, 2, 3, 4, 7, 10, and 14 post-exercise. Plasma CK and LDH activity significantly increased during the days post-exercise, and remained elevated above baseline until day 10 post-exercise. The time course and magnitude of increased CK and LDH in plasma following the resistance exercise session was in accordance with previous work [7,35], with maximum CK and LDH activity occurring approximately 72 to 96 hours after the resistance exercise. The delay in maximal elevation of CK and LDH activity is most likely caused by the increasing membrane permeability due to secondary or delayed onset damage as a result of increasing Ca^2+ ^leakage into the muscle [36]. Additionally, the differences in the magnitude of CK and LDH present in plasma following eccentric exercise (as seen in Figures 4 and 5) is possibly due to the larger molecular weight of LDH compared to CK and hence a decreased ability to diffuse from the muscle cell following injury [37].

In the current study, plasma CK activity was significantly lower (~84% on average) at day 2, 3, 4, and 7 in the Cr-CHO supplemented group compared to the CHO group following exercise-induced muscle damage, with a similar trend (~12% lower) in LDH activity. Less leakage of muscle enzymes from the muscle may be indicative of less damage to the muscle, which may be due to improved Ca^2+ ^buffering capacity of the muscle (i.e. the rate of Ca^2+ ^removal from the muscle cytoplasm) and thus less Ca^2+ ^accumulation within the cell and subsequent proteolytic activation.

In summary, the major finding of this investigation was significantly higher muscle strength after Cr supplementation during recovery from a muscle damaging exercise session. While this may be due in part to a faster muscle growth during the recovery period, significantly lower plasma creatine kinase activity in the days after injury is indicative of less muscle damage.

Perspective

It is clear that a limitation to the current study is that the exact mechanisms by which Cr exerts its effects were not examined, and thus, further research is needed. However, it is evident from other studies that Cr is perhaps working via two possible mechanisms in the current study. Firstly, Cr supplementation prior to eccentric-induced damage may be enhancing the calcium buffering capacity of the muscle by fuelling the SR Ca^2+^-ATPase pump, thereby decreasing intracellular calcium concentrations and activation of degradative pathways such as calpain. Thus, a reduction in calcium-activated proteases will minimise additional damage to the sarcolemma, but more importantly, further influxes of calcium into the muscle. Secondly, Cr supplementation post-exercise may enhance one or more of the key phases involved in the regenerative response to exercise-induced damage, such as increasing protein synthesis, reducing protein degradation, and thus, creating an environment that facilitates enhanced satellite proliferation and hence formation of new muscle fibres. This combination is likely to allow a higher training volume to be maintained during subsequent exercise sessions during resistance training.

## Competing interests

This study was funded by AST Sports Science Pty Ltd (USA) through an unrestricted research grant to Victoria University.

## Authors' contributions

MC was the study coordinator and was involved in data analysis and manuscript preparation. ER and AW assisted in data collection. PC assisted in data collection, research design and obtaining grant funding. AH was involved in research design, grant funding, manuscript preparation and PI of the study.
